# The Impact of Dietary Consumption of Palm Oil and Olive Oil on Lipid Profile and Hepatocyte Injury in Hypercholesterolemic Rats

**DOI:** 10.3390/ph15091103

**Published:** 2022-09-04

**Authors:** Tarfa Albrahim, Maram H. M. Alotaibi, Norah M. M. Altamimi, Atheer M. A. Albariqi, Lamees A. O. Alqarni, Sara N. A. Alassaf, Hisham S. Aloudah, Mohammed Alahmed, Ahmad T. Almnaizel, Maha R. Aldraihem, Mona Alonazi

**Affiliations:** 1 Department of Health Sciences, Clinical Nutrition, College of Health and Rehabilitation Sciences, Princess Nourah Bint Abdulrahman University, P.O. Box 84428, Riyadh 11671, Saudi Arabia; 2 Prince Naif for Health Research Center, King Saud University, Riyadh 11472, Saudi Arabia; 3 Department of Biochemistry, College of Science, King Saud University, Riyadh 11451, Saudi Arabia

**Keywords:** palm oil, olive oil, hypercholesterolemia, oxidative stress, inflammation, FAS

## Abstract

A metabolic disease called hypercholesterolemia is connected to both oxidative damage and inflammation. The goal of the current investigation was to determine if olive oil and palm oil could prevent hypercholesterolemia-induced oxidative stress in the liver of rats fed a high-cholesterol diet (HCD). The experimental mice were given HCD for three months while also receiving 0.5 mL/kg of either palm or olive oil. Serum triglycerides, total cholesterol, LDL cholesterol, vLDL cholesterol, and the atherogenic index all significantly increased in HCD-fed rats, while HDL cholesterol significantly dropped. Additionally, HCD caused a notable rise in proinflammatory cytokines and serum transaminases in liver tissue. Additionally, HCD significantly increased the production of nitric oxide and lipid peroxidation in the liver while decreasing antioxidant enzymes. Treatment with palm and olive oils dramatically reduced the levels of pro-inflammatory cytokines and lipid peroxidation, improved antioxidant defenses, and considerably improved liver function indicators. Additionally, the examined oils dramatically decreased the expression of fatty acid synthase (FAS) in the liver of rats receiving HCD. In conclusion, HCD-fed rats exhibit significant antihyperlipidemic and cholesterol-lowering benefits from palm and olive oils. The improved antioxidant defenses, lower inflammation and lipid peroxidation, and altered hepatic FAS mRNA expression were the main mechanisms by which palm and olive oils produced their advantageous effects.

## 1. Introduction

Lipid intake that is not controlled can result in a life-threatening condition. Hypercholesterolemia is defined as a group of non-communicable disorders. Primary (genetic or familial) or secondary (acquired) causes are both possible [1]. High lipid intake is seen as a serious public health issue that has a negative impact on people’s quality of life and health, with a prevalence of 20% to 44% among adults in Saudi Arabia [2]. Cardiovascular disease (CVD), hypertension (HT), and type 2 diabetes mellitus (T2DM) are all closely connected to hypercholesterolemia [3]. Unhealthy diets, especially high dietary fat intake, are the most important behavioral risk factors for heart disease and stroke, according to World Health Organization (WHO) statistics from 2019. As a result, CVD has surpassed cancer as the top cause of death worldwide, killing 17.9 million people each year, with one-third of these deaths occurring before the age of 70 [4].

Lipids are divided into two types, saturated fatty acids (monounsaturated fatty acids and polyunsaturated fatty acids) and unsaturated fatty acids (monounsaturated fatty acids and polyunsaturated fatty acids). Each form of lipid has its own digestive, transport, storage, and metabolic pathways, as well as impacts on the body. Mba et al. [5] revealed in a research review conducted in 2012 that palm oil (PO) was the primary oil source around the world, accounting for 32 percent of total worldwide oil production. Palmitic acid in palm oil has recently been the focus of attention about its effects on CVD and coronary heart disease, owing to the widespread consumption of foods high in saturated fatty acids, notably palm oil [3]. Olive oil (OO), on the other hand, is classified as monounsaturated fatty acids (MUFAs). A decreased frequency of CVD and normal cholesterol levels has been reported in the Mediterranean region, which has been linked in part to the Mediterranean diet’s regular consumption of olive oil [3]. Consuming OO may protect people from developing metabolic syndrome, type II diabetes, obesity, many cancers, cardiovascular disorders, and other illnesses. Moreover, healthy people have reported that OO has positive impacts [6]. The two main components of OO are phenolic antioxidants and fatty acids. Vitamin E, oleocanthal, carotenoids, and polyphenols, including hydroxytyrosol, hydroxytyrosol acetate, and oleuropein are just a few of the many antioxidants found in OO [7]. Oleuropein in particular reduces the oxidation of low-density lipoprotein particles [6]. Palm oil, on the other hand, has been widely used for cooking throughout West Africa and Asia for over a thousand years. Nowadays, there is considerable confusion in western countries about the term “palm oil” because of the percentage that is currently used in food (cakes, puff pastries, biscuits, fat spreads, and so on), because palm oil has replaced trans fatty acid-rich hydrogenated lipids in such foods [3]. Folklore medicine views palm oil as a treatment for headaches, pain, rheumatism, thrombosis, and cardiac conditions. It has been reported to have antidotal, aphrodisiac, and diuretic properties [8].

Obesity and hyperlipidemia have become a global epidemic as a result of a shift toward a high-fat diet, particularly one heavy in saturated fat. As a result, hyperlipidemia and obesity are on the rise in Saudi Arabia, with increased prevalence rates. According to an article published by Althumiri et al. in KSA [1], the prevalence of obesity weighing more than 30 BMI (BMI > 30) was 24.7 percent. Another study found that the overall prevalence of dyslipidaemia in KSA ranged from roughly 20% to 40%, which is consistent with the previous data. However, triglycerides had the highest frequency in persons with dyslipidaemia, at around 44%, followed by hypercholesterolemia [2].

The effects of palm oil and olive oil on serum lipid profiles are still debated. As a result, more research is needed to fully comprehend the effects of palm oil and olive oil on hypercholesterolemic lipid profiles and lipoproteins. The goal of this study is to see how dietary consumption of palm oil and olive oil affects the serum lipid profiles of rats in hypercholesterolemic models. The goal of this study is to see how dietary palm oil and olive oil affect hypercholesterolemic rats’ biochemical blood lipid profiles.

## 2. Results

### 2.1. GC-MS Results of Olive and Palm Oils

Table 1 displays the respective percentage areas and retention time of the olive oil investigated fatty acids and fatty acid methyl esters. Hexadecanoic acid methyl ester, hexadecanoic acid (palmitic acid), 9,12,15-octadecatrienoic acid (alpha-linolenic acid), (Z)-9-octadecenoic acid methyl ester, 9-octadecenoic acid (oleic acid), and vitamin E were the primary constituents of olive oil as determined by the GC-MS (Supplementary data; Figure S1).

Table 2 displays the respective percentage areas and retention time of the palm oil investigated fatty acids and fatty acid methyl esters. Mainly, the oil contained palmitic and oleic acids. In addition, the oil contained squalene and ethyl iso-allocholate. The primary constituents of palm oil as determined by the GC-MS was provided in Supplementary data (Figure S2).

### 2.2. Effect of Palm and Olive Oils on Body Weight, Blood Glucose Level, and Liver Function Enzymes

**Figure 1:**
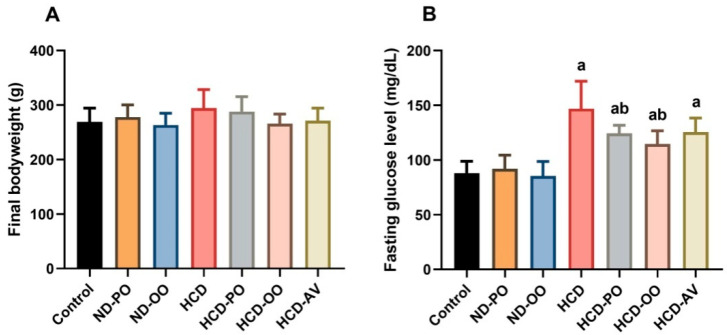
(**A**) Body weight and (**B**) serum glucose level following treatment with palm oil (PO), olive oil (OO), or atorvastatin (AV) in high-cholesterol diet-induced hypercholesterolemia in rats. Data are expressed as the mean ± SD (*n* = 7). Letters a and b indicate statistically significant differences between control rats and hypercholesterolemia rats, respectively, at *p* < 0.05.

Feeding on a high-cholesterol diet did not alter the body weight of rats significantly (Figure 1A). However, co-administration of PO or OO caused a decrease in the body weight of rats fed on HCD. The decrease in body weight in those groups, HCD-PO and HCD-OO, was not significantly (*p* > 0.05) different from either control or HCD-fed rats.

Obvious elevation (*p* < 0.05) was found in serum glucose levels in rats submitted to HCD when compared with those fed on a normal diet (Figure 1B). On the other hand, hypercholesterolemia rats supplemented with PO or OO displayed significant diminishment (*p* < 0.05) in fasting glucose levels. However, HCD-fed rats that received AV displayed lower levels of glucose, but this decrease was not significant compared with HCD-fed rats (Figure 1B).

**Figure 2:**
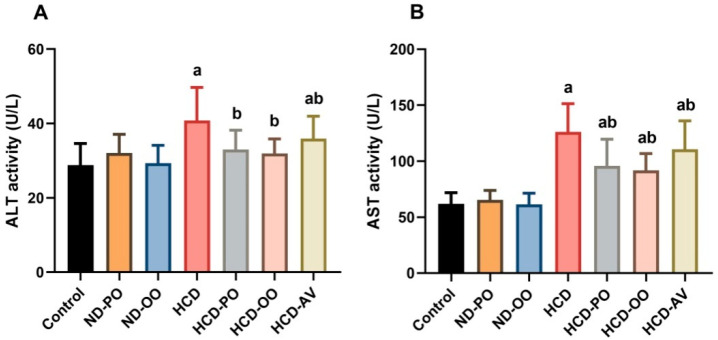
Serum transaminases ((**A**) ALT and (**B**) AST) following treatment with palm oil (PO), olive oil (OO), or atorvastatin (AV) in high-cholesterol diet-induced hypercholesterolemia in rats. Data are expressed as the mean ± SD (*n* = 7). Letters a and b indicate statistically significant differences between control rats and hypercholesterolemia rats, respectively, at *p* < 0.05.

Rats fed on HCD displayed notable elevations (*p* < 0.05) in serum levels ALT and AST when compared to those that received a normal diet. However, supplementation of rats with OO, PO, or AV evoked marked decreases (*p* < 0.05) in serum levels of hepatic function markers compared with the HCD-supplemented group (Figure 2).

### 2.3. Effect of Palm and Olive Oils on Lipid Profile

**Figure 3:**
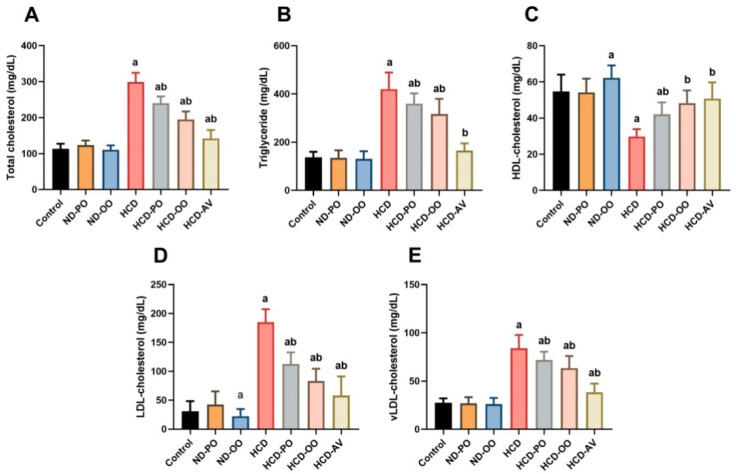
Serum levels of (**A**) triglyceride, (**B**) cholesterol, (**C**) HDL, (**D**) LDL, and (**E**) vLDL following treatment with palm oil (PO), olive oil (OO), or atorvastatin (AV) in high-cholesterol diet-induced hypercholesterolemia in rats. Data are expressed as the mean ± SD (*n* = 7). Letters a and b indicate statistically significant differences between control rats and hypercholesterolemia rats, respectively, at *p* < 0.05.

Noticeable increases (*p* < 0.05) were exhibited in TG, TC, LDL-c, and vLDL-c, accompanied by significant declines in HDL-c levels in rats fed on HCD when compared with those fed on a normal diet. On the other hand, the hypercholesterolemic group supplemented with OO, PO, or AV displayed considerable decreases (*p* < 0.05) in TG, TC, and LDL-c, along with elevations in HDL-c levels (Figure 3).

### 2.4. Effect of Palm and Olive Oils on Atherogenic Index and Fatty Acid Synthase (FAS) Expression

**Figure 4:**
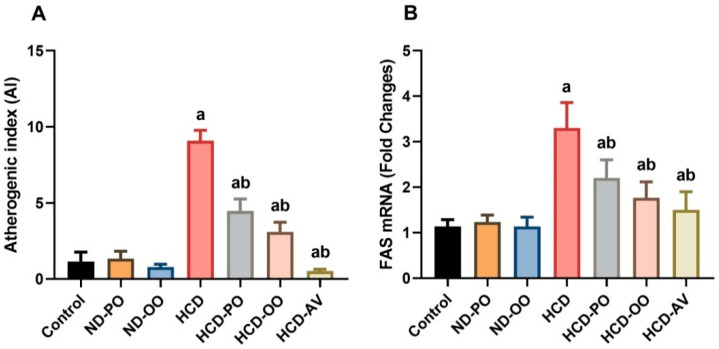
(**A**) Atherogenic index and (**B**) fatty acid synthase expression following treatment with palm oil (PO), olive oil (OO), or atorvastatin (AV) in high-cholesterol diet-induced hypercholesterolemia in rats.Data are expressed as the mean ± SD (*n* = 7). Letters a and b indicate statistically significant differences between control rats and hypercholesterolemia rats, respectively, at *p* < 0.05.

Significant increases (*p* < 0.05) were recorded in the atherogenic index in rats submitted to HCD when compared to those fed on a normal diet (Figure 4A). In contrast, hypercholesterolemic rats that received OO or PO provoked significant lessening (*p* < 0.05) of the AL relative to rats fed on HCD. As expected, AV administration significantly decreased AL compared to both control and HCD-fed rats.

Hepatic FAS was assessed in hypercholesterolemic rats with or without PO or OO supplementation. Significant upregulation (*p* < 0.05) was observed in FAS expression in the group that received HCD in respect to the control rats. However, PO, OO, or AV administration promoted a marked anti-adipogenesis effect as witnessed by downregulating (*p* < 0.05) of FAS expression in hepatic tissue (Figure 4B).

### 2.5. Effect of Palm and Olive Oils on Hepatic Oxidative/Antioxidant Status

**Figure 5:**
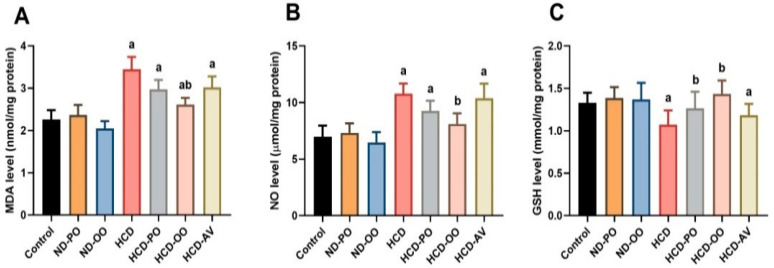
Hepatic levels of oxidative stress markers ((**A**) lipid peroxidation, (**B**) nitric oxide, and (**C**) glutathione) following treatment with palm oil (PO), olive oil (OO), or atorvastatin (AV) in high-cholesterol diet-induced hypercholesterolemia in rats.Data are expressed as the mean ± SD (*n* = 7). Letters a and b indicate statistically significant differences between control rats and hypercholesterolemia rats, respectively, at *p* < 0.05.

It is clear from Figure 5 that the HCD-fed rats unveiled marked elevations (*p* < 0.05) in hepatic LPO and NO levels accompanied by a significant decrease in GSH content when compared with the normal diet-fed group. Nevertheless, noteworthy declines in LPO and NO levels and a significant increase in GSH content were detected in groups that received PO, OO, or AV treatment in comparison with the hypercholesterolemic group.

**Figure 6:**
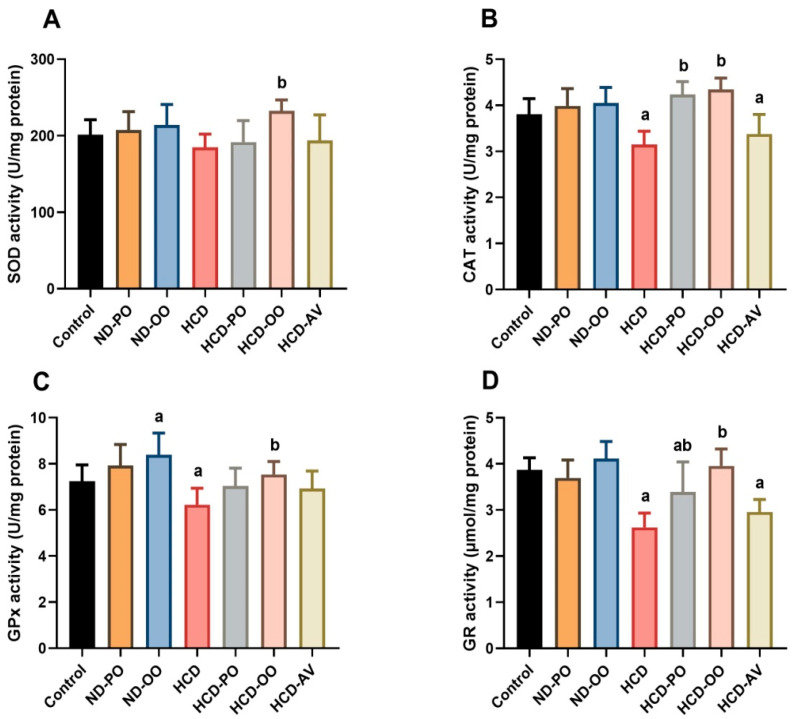
Hepatic levels of oxidative stress markers ((**A**) superoxide dismutase, (**B**) catalase, (**C**) glutathione peroxidase, and (**D**) glutathione reductase) following treatment with palm oil (PO), olive oil (OO), or atorvastatin (AV) in high-cholesterol diet-induced hypercholesterolemia in rats.Data are expressed as the mean ± SD (*n* = 7). Letters a and b indicate statistically significant differences between control rats and hypercholesterolemia rats, respectively, at *p* < 0.05.

As shown in Figure 6, there were significant decreases (*p* < 0.05) in enzymatic activities of SOD, CAT, GR, and GPx in the livers of hypercholesterolemic rats when compared to the normal control group. However, OO, PO, or AV administration to hypercholesterolemic rats prompted a marked antioxidant effect as witnessed by increases (*p* < 0.05) in levels of SOD, GR, and CAT.

### 2.6. Effect of Palm and Olive OILS on Hepatic Inflammatory Biomarkers

**Figure 7:**
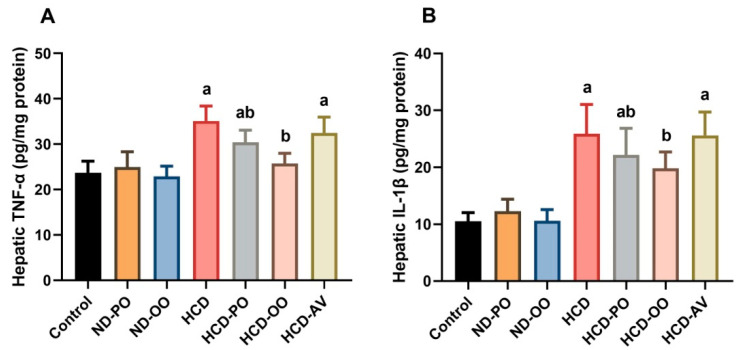
Hepatic levels of pro-inflammatory cytokines ((**A**) IL-1β and (**B**) TNF-α) following treatment with palm oil (PO), olive oil (OO), or atorvastatin (AV) in high-cholesterol diet-induced hypercholesterolemia in rats. Data are expressed as the mean ± SD (*n* = 7). Letters a and b indicate statistically significant differences between control rats and hypercholesterolemia rats, respectively, at *p* < 0.05.

Levels of hepatic IL-1β and TNF-α exhibited notable increases (*p* < 0.05) in the HCD-administered group compared to the group fed on a normal diet. However, OO, PO, or AV administration to hypercholesterolemic rats meaningfully ameliorated (*p* < 0.05) the levels of tested pro-inflammatory cytokines in hepatic tissues (Figure 7).

## 3. Discussion

Health specialists around the world have severe concerns about the risk of excessive cholesterol buildup inside the body and cardiovascular disorders in the twenty-first century. With no negative side effects, olive oil has been used for a long time to promote cardiovascular health under cellular and environmental stress and to lower the risk of cardiovascular events and cardiovascular death [9,10]. However, there is controversy around the use of palm oil as edible oil because it contains palmitic acid, a saturated fatty acid that should logically result in higher levels of total cholesterol and low-density lipoprotein cholesterol. To explore the biological benefit of PO, numerous research studies in both humans and animals have conclusively demonstrated that consuming palm oil does not result in increased serum levels of cholesterol and that palm oil is not atherogenic. In addition to palmitic acid, oleic and linoleic acids, which are monounsaturated and polyunsaturated, respectively, are also found in palm oil [11]. Additionally, palm oil contains vitamins A and E, two potent antioxidants. Scientific research has demonstrated that palm oil can shield blood vessels and the heart from ischemia damage and plaque buildup [12]. As a result, numerous investigations on the cardioprotective properties of OO and PO were conducted. On the other hand, little is known about how OO and PO affect hypercholesterolemia.

Rats receiving HCD for three months displayed dyslipidemia and hypercholesterolemia, as evidenced by the large rises in TG, TC, LDL-c, and vLDL-c serum levels together with significant drops in HDL-c levels. These outcomes were consistent with research showing that rats fed HCD developed hypercholesterolemia [13,14]. Regardless of the cause, dyslipidemia and hypercholesterolemia share the common trait of a disturbed serum lipid profile. Increased hepatic lipid accumulation brought on by dyslipidemia may limit the liver’s capacity to lower levels of these lipid constituents [15]. Hepatic cell damage results from increased hepatic lipid buildup in hypercholesterolemia instances [15]. In the current study, rats given HCD had significantly higher serum levels of ALT and AST, which resulted in liver damage. Interestingly, serum lipids and liver function markers significantly improved in HCD-fed rats treated with PO or OO. These findings support the studied oils have hepatoprotective and anti-hypercholesterolemic properties.

In this regard, the Mediterranean diet has been linked to lower levels of non-high-density lipoprotein cholesterol (non-HDL-C) and atherogenic cholesterol LDL-C [16]. In their study, Hernáez et al. [16] demonstrated that 1 year of intervention with a Mediterranean diet, particularly when enhanced with virgin olive oil, improves several HDL functions in people at high cardiovascular risk, including cholesterol efflux capacity, cholesterol metabolism, antioxidant/anti-inflammatory properties, and vasodilatory capacity. Similar findings were made by Covas et al. [17], who discovered that consumption of phenolic olive oils enhanced HDL-C while decreasing the TC/HDL-C ratio, LDL-C/HDL-C ratio, and triglyceride levels.

Administration of PO did not significantly alter the lipid profile in the current investigation. This is in line with the previous study, which found that consuming PO and palm olein had no negative impacts on the lipoprotein profiles. Through the pool of nine studies that were chosen, no study indicated a noticeably higher serum or plasma TC concentration in comparison to the control group. However, two studies showed that consuming PO and palm olein was safe because TG levels were significantly reduced, which highlights the potential of this oil to treat hypertriglyceridemia [18]. In addition, numerous studies showed that PO or palm olein might considerably diminish serum or plasma LDL-C concentrations [18,19,20].

The capacity of PO and OO to reduce the expression of the FAS gene in the rat liver may be responsible for the reported lipid-lowering impact seen in this investigation. One of the fatty acid-synthesizing enzymes, FAS, has been linked to increased liver expression in rodents fed high-fat or high-cholesterol diets in numerous studies [14,15]. According to Lee et al. [15] findings, HCD-induced rats in the current study displayed a considerable increase in the gene expression of FAS. In our study, we found that PO and OO reduce the expression of hepatic FAS to achieve their lipid-lowering effects. The investigated oils’ advantageous effects on rats receiving HCD may be attributed to their active ingredients. Khatun et al. [21] reported down-regulated expressions of FAS when broilers were given increased doses of palm oil, demonstrating the ability of PO to downregulate FAS. Because palmitic acid is thought to be hypercholesterolemic, this decrease in concentration with oil supplementation was significant. Therefore, a diet high in palmitic acid (palm oil) supplementation appears to down-regulate the synthesis of new fatty acids, lowering the concentration of the resulting palmitic acid, a fatty acid that is hypercholesterolemic. The ability of OO in restraining the FAS expression was reported previously by many researchers [22,23]. Normal rat hepatocytes exposed to single phenols, such as hydroxytyrosol, tyrosol, and oleuropein [22], or exposed to an OO-high phenol extract [23], showed a downregulation of fatty acid, cholesterol, and triglyceride synthesis. Notably, the combined effects of the investigated phenols [22] are less potent than the individual effects on hepatic lipid production, which suggests a synergistic activity of the phenols present in the OO extract [23].

Cholesterol rich diets led to oxidative stress in the liver tissue in the current investigation, which was demonstrated by a large rise in LPO and NO levels and a considerable fall in GSH and antioxidant enzyme activity. Changes in lipids play a role in the development of oxidative stress in obese individuals. An imbalance between oxidant–antioxidant activity and ROS is a characteristic of lipid peroxidation, which is regarded to be a disease brought on by obesity [14,24]. Hyperlipidemia can cause oxidative stress, which can cause damage and cell death. It can also cause decreased antioxidant defaces and increased lipid peroxidation. As a result, prior research has shown that obesity and hyperlipidemia are separate risk factors for rising lipid peroxidation, declining cytoprotective mechanisms, and cell death [25]. In addition to lipid peroxidation, nitric oxide (NO), a powerful vasodilator and anti-aggregating molecule, is inactivated by ROS [26]. Endothelial NO availability is significantly impacted by changes in endothelial redox status. At the same time, hypercholesterolemia stimulated inducible nitric oxide synthase (iNOS) that subsequently increased NO production results in cytotoxicity via reacting with superoxide (O_2_.-) to form the potent oxidant peroxynitrite (ONOO−) [27].

However, OO or PO has the ability to restrain oxidative stress in liver tissue of HCD-fed rats. According to previous research [8], palm oil’s crude fractions and gel fractions both contain significant levels of tocopherols and carotenoids, which may account for their efficacy in preventing lipid peroxidation. Due to the abundant availability of phospholipids, which interact with vitamin E and other antioxidant cascades for the maintenance of oxidative equilibrium, these phytonutrients have also been linked to numerous instances of tissue fortification. This is also consistent with the submissions of Twumasi et al. [28], who noted that these extracts had the ability to restore redox status after the oil had been used to treat rats poisoned with lead. Evidence from the literature demonstrating the protective effects of palm oil-supplemented diets against heart disease, tissue damage, and myocardial infarction also supports the argument for the effectiveness of palm oil and palm oil fractions to modify the induction of myocardial infarction [12,29]. Giving validity to our prior assertions, documented increases in MDA values relative to the control group coincide with higher depletion of hepatic and renal GSH, CAT, and SOD compared to the control group in all post-cadmium dosing periods. Asagba and Eriyamremu’s earlier study from [30] on the positively linked rise in MDA values and contemporaneous decline in SOD, CAT, and ATPase activity after oral cadmium consumption lends support to this.

The “minor” components of palm oil are what give it its nutritional advantages, which are related to its antioxidant power [31]. Numerous research studies on animals have emphasized the antioxidant properties of PO tocotrienols. Coenzyme Q10 (ubiquinone), a naturally occurring coenzyme of PO, has ten times the antioxidant potential of carotenoids and vitamin E and is a potent free radical scavenger. The vegetable oil with the highest concentration of tocotrienols is palm oil. Tocotrienols are potent antioxidants with a known cardioprotective effect [32]. The findings of this study demonstrate that PO still retains its antioxidant effects despite the loss of carotenoids and micro-constituents including flavonoids and phenolic acids after refining. This might be because tocotrienols, potent free radical scavengers, are added to PO during refining, enhancing their antioxidant properties [31].

The concept that chronic inflammation contributes to the onset and progression of atherosclerosis is now well acknowledged. Chronic inflammation has recently been identified as a contributory element in the development of several chronic illnesses, including CVD. T-cells, monocytes, and neutrophils are a few examples of inflammatory cell types that play significant roles in mediating the inflammatory response associated with atherosclerosis [33]. When lipid and lipoproteins are maintained in the arterial wall, especially when the lipids are oxidatively damaged, the inflammatory response starts. Foam cells are created when these lipids and lipoproteins are absorbed by macrophages, dendritic cells, and smooth muscle cells. The inflammatory response in the vascular wall is further elaborated by the generation and release of proinflammatory cytokines and chemokines, such as tumor necrosis factor (TNF), interferons (IFNs), monocyte chemoattractant protein-1, stromal cell-derived factor-1, and macrophage inflammatory protein-1. Oxidative stress and its pro-inflammatory condition within the atherosclerotic lesion both speed up atheroprogression [34]. Interestingly, pro-inflammatory markers in liver tissues significantly improved in HCD-fed rats treated with PO or OO. These findings support the studied oils have an anti-inflammatory property.

In the present study, OO administration showed a significant anti-inflammatory effect against HCD in liver tissue. A further significant mechanism that may help to explain the association between OO and the development of cardiovascular disease is its anti-inflammatory action on the arterial wall. In their work, Fki et al. [35] assessed the levels of inflammatory markers such COX-2, NF-κB, and TNF-α, and found that oleuropein- and hydroxytyrosol-rich olive leaf extracts possessed hypolipidemic and hepatoprotective effects against the high-fat diet-induced metabolic disorders by enhancing the antioxidative defense system and blocking the expression of the proteins involved in inflammation and liver damage. Moreover, the anti-inflammatory activities of hydroxytyrosol including the inhibition of both myeloperoxidase enzyme (MPO) and pro-inflammatory cytokines such as IL-1 and IL-6 in mice [36]. Furthermore, in patients with high cardiovascular risk who adhered to the Mediterranean diet supplemented with OO, these indicators improved, indicating a delay in the production of atheroma plaques [37,38,39]. Indeed, OO polyphenols have strong anti-inflammatory effects as stated by others. In this regard, Al-Quraishy et al. [40] found that olive leaf extract attenuated the inflammatory response by decreasing NF-κB, COX-2, and TNF-α expressions and down-regulating iNOS and IL-1β in gastric mucosa against HCl/ethanol-induced gastric mucosal damage in rats. Additionally, Yin et al. [41] reported that oleuropein restrained pro-inflammatory mediators in renal tissue of glycerol-induced acute kidney injury (AKI) in rats.

In the present study also, PO showed a good anti-inflammatory effect against HCD. By having a high tocotrienol concentration, PO could explain how it affects the pro-inflammatory cytokines. Tocotrienols’ role in preventing the NF-κB activation pathway contributes to their anti-inflammatory effects. Studies conducted in vitro with PO tocotrienols have demonstrated their anti-inflammatory properties and their capacity to decrease cancer cell growth by blocking the NF-κB activation pathway [42]. Animal studies have demonstrated that diets high in PO and OO do not increase inflammation in the plasma [31,43]. The findings of this study and information from the literature demonstrate that PO has anti-inflammatory properties because of its high tocotrienol content, despite its abundance in saturated fatty acids, particularly palmitic acid, which is particularly pro-inflammatory because it activates the NF-B pathway [31].

## 4. Materials and Methods

### 4.1. GC-MS Analysis

Olive oil and palm oil were purchased from a local market in Riyadh, KSA, and then were subjected for GC-MS analysis for identification of different constituents. The GC-MS analysis was performed using Shimadzu GC-2010 Plus. Agilent J&W DB-5ms (5%-phenyl)-methylpolysiloxane capillary column (30 m × 2.5 mm; 0.25 mm film thickness) was employed. The injection port temperature was kept at 250 °C while the transfer line and ion source temperatures were maintained at 240 °C. Helium gas was used as a carrier with a flow rate of 1 mL min^−1^. The analysis was performed with the following temperature program: 1 min. of isothermal heating at 100 °C followed by heating at 300 °C for 20 min. The ionizing energy was 70 eV. The mass spectra were recorded, and each component was quantified based on peak areas and normalization based on the internal standard.

### 4.2. Animals and Ethical Statement

Male Wistar rats (weighing approximately 200–220 g) were housed under a controlled environmental temperature (25 ± 1 °C), humidity (50 ± 10%), and lighting (12 h light/dark cycle) condition. Prior to experimentation, rats were acclimated for two weeks with free allowance for water and food. All the experimental procedures followed the guidelines of the Institutional Animal Care and Use Committee (IACUC) of Princess Nourah Bint Abdulrahman University (Approval No. HAP-01-R-059; IRB Registration No. 21-0502; Category of Approval: EXEMPT).

### 4.3. Experimental Design

After being acclimated, rats were allocated to 7 groups (*n* = 7 per each group) as follows: the first group (Control) served as control and fed on a normal basal diet until the end of the experiment. The second and third groups were supplemented with a normal basal diet until the end of the experiment, concurrently; the rats of those groups were administered olive oil at 0.5 mL/kg bodyweight and palm oil at 0.5 mL/kg bodyweight and served as ND-OO and ND-PO, respectively. The fourth group (HCD) was supplemented with a normal basal diet containing cholesterol (1%; *w*/*w*). The cholesterol batches were mixed carefully with the basal diets just before the diets were offered to the rats [13]. The fifth, sixth, and seventh groups were fed on a normal basal diet containing cholesterol (1%; *w*/*w*) and administered palm oil (HCD-PO; 0.5 mL/kg bodyweight), olive oil (HCD-OO; 0.5 mL/kg bodyweight), or atorvastatin (HCD-AV; 10 mg/kg bodyweight) orally every day. The weights of all rats from different groups were recorded during the experiment to calculate the bodyweights and to adjust the administered dose in order to ensure a constant dose.

### 4.4. Sampling and Tissue Preparation

Six hours prior to animal euthanizing, following an intervention period of 3 months, all food was removed from cages for all groups, while the animals were provided water as normal. Rats were euthanized by an overdose of pentobarbital (300 mg/kg, i.p.). The blood was immediately collected, left for 30 min at 37 °C, and centrifuged at 3000× *g* for 10 min for serum separation that was stored at −80 °C for biochemical analysis. The liver was collected immediately. The isolated liver tissue was homogenized with 10 times volume of ice cold 50 mM potassium phosphate buffer (pH 7.4) and homogenized. The supernatant was separated by centrifugation at 3000× *g* (4 °C) for 10 min. The supernatants were stored at −80 °C and used for biochemical analysis. Some parts of the liver tissues were sampled and stored at −80 °C until gene expression analyses or fixed in 10% buffered formalin for histopathological examinations.

### 4.5. Serum Biochemical Parameters

Blood glucose levels were assessed using an available enzyme colorimetric assay kit according to Trinder [44]. Total cholesterol (TC), total triglycerides (TG), and high-density lipoprotein (HDL-c) were measured using commercial kits from RANDOX Reagents (USA). LDL conc. can be determined via the following equation (Friedewald formula [45]): LDL-c = TC − (HDL-c + TG/5). For calculating very LDL (vLDL), the Sampson et al. [46] equation was used as the following: vLDL = TG/5. Additionally, alanine transaminase (ALT) and aspartate transaminase (AST) were measured for the assessment of liver function according to the manufacturer’s instructions (RANDOX Reagents; Crumlin, UK).

### 4.6. Atherogenic Index

Determination of the atherogenic index (AI) was calculated based on both the ratio between serum levels of LDL-c and HDL-c, and the ratio between triglyceride levels and HDL-c, according to the method of Kazemi et al. [47]

### 4.7. Oxidative Stress Markers

To assess biomarkers for oxidative stress, malondialdehyde (MDA) and nitric oxide (NO) levels were measured based on the protocol of Ohkawa et al. [48] and Griess reagent [49], respectively. In addition, the reduced glutathione (GSH) contents were measured following the method of Ellman [50]. Briefly, 0.5 mL of liver homogenate and 1 mL each of trichloroacetic acid (1:10) and thiobarbituric acid (0.67%) were heated in a water bath for 30 minutes. By measuring absorbance at 535 nm, thiobarbituric acid reactive compounds were quantified as the amount of MDA produced. However, the optimal acid reduction method in an acidic medium and with nitrite was used to measure the amount of nitric oxide in the hepatic tissue. The resultant brilliant reddish purple azo dye can be detected at 540 nm when nitrous acid diazotized sulfanilamide and N-(1-naphthyl) ethylenediamine are combined in this synthesis. By reducing 5,5′-dithiobis (2-nitrobenzoic acid) (Ellman’s reagent) with GSH to generate a yellow molecule, the amount of hepatic glutathione (GSH) was ascertained. The amount of reduced chromogen is directly proportional to the GSH concentration, and its absorbance can be measured at 405 nm. Whereas, vitamin-E (tocopherol) was estimated according to the previous reported method [51].

### 4.8. Antioxidant Enzymatic Activities

The methods described by Nishikimi et al. [52] and Aebi [53] were utilized for assessment of superoxide dismutase (SOD) and catalase (CAT) activities, correspondingly. Further, the activities of glutathione peroxidase (GPx) were determined according to Paglia and Valentine [54] and glutathione reductase (GR) was estimated based on the methods of De Vega et al. [55]

### 4.9. Determination of Inflammatory Markers

The levels of interleukin-1β (IL-1β) and tumor necrosis factor-α (TNF-α) in liver tissue were measured by ELISA kits obtained from R&D Systems (Minneapolis, MN, USA) according to the manufacturer’s instructions.

### 4.10. RNA Extraction, cDNA Synthesis and Quantitative RT-PCR Analysis

TRIzol reagent (Qiagen, Germantown, MD, USA) was used to extract total RNA from freshly isolated liver tissues according to the manufacturer’s instructions. Using the RevertAid H Minus Reverse Transcriptase kit (Fermentas, Thermo Fisher Scientific Inc., Waltham, MA, USA), cDNA was produced in accordance with the manufacturer’s instructions after RNA concentrations were determined using nanodrop. FAS mRNA levels were determined using a SYBR green PCR kit from Qiagen in Hilden, North Rhine-Westphalia, Germany. On a ViiATM 7 PCR instrument, quantitative PCR was conducted twice (Applied Biosystems, Waltham, MA, USA). The 2Ct technique, which was standardized to the mRNA level of the GAPDH housekeeping gene, was used to determine the relative amounts of FAS mRNA. The primer sequence for FAS was forward 5′-tgtaccctctagctggaccc-3′ and reverse 5′-ccaggctaagggcaatggaa-3′, and the primers sequence for glyceraldehyde-3-phosphate dehydrogenase (GAPDH) were forward 5′-agtgccagcctcgtctcata-3′ and reverse 5′-accagcttcccattctcagc-3′.

### 4.11. Statistical Analysis

All data were statistically analyzed using SPSS software. The one-way ANOVA was used to assess the significant difference between different groups followed by the Turkey’s post hoc test. The obtained data were displayed as the mean ± SD. *p* value less than 0.05 was considered to be significant.

## 5. Conclusions

The present study revealed that palm oil and olive oil have potent antioxidant and cholesterol-lowering effects on HCD-fed rats. PO and OO significantly diminished serum lipid markers, pro-inflammatory cytokines, and anti-apoptotic proteins. The lipid-lowering effect of PO and OO were mediated partially through the restrained hepatic FAS expression. However, further studies are recommended to determine the exact lipid-lowering mechanisms of PO and OO.

## Figures and Tables

**Table 1:** Fatty acids and fatty acid methyl esters molecular formula, RT, and area (%) in olive oil.

Peak Number	Compounds	Molecular Formula	RT	Area %
1	Hexadecane	C_17_H_36_	8.18	1.19
2	Tetradecane, 2,6,10-trimethyl-	C_21_H_44_	12.57	2.63
3	Docosane	C_22_H_46_	13.46	3.90
4	9,12,15-Octadecatrienoic acid (alpha-Linolenic acid)	C₁₈H₃₀O₂	15.86	7.31
5	Dotriacontane	C_32_H_66_	17.36	1.52
6	Hexadecanoic acid, methyl ester	C_17_H_34_O_2_	20.20	6.09
7	Pentadecanoic acid, 14-methyl-, methyl ester	C_17_H_34_O_2_	20.90	1.17
8	Hexadecanoic acid (Palmitic acid)	C_16_H_32_O_2_	22.20	13.05
9	9-Octadecenoic acid (Z)-, methyl ester	C_19_H_36_O_2_	23.20	27.33
10	2,2-Dideutero octadecanal	C_19_H_26_O_6_	24.12	0.83
11	9-Octadecenoic acid (Oleic acid)	C_18_H_34_O_2_	24.67	11.25
12	2,6,10,14,18,22-Tetracosahexaene	C_30_H_50_	32.56	7.81
13	Ethyl iso-allocholate	C_26_H_44_O_5_	33.68	5.26
14	Vitamin E	C_29_H_50_O_2_	34.56	5.15
15	β-sitosterol	C_29_H_50_O	35.43	5.51

**Table 2:** Fatty acids and fatty acid methyl esters molecular formula, RT, and area (%) in palm oil.

Peak Number	Compounds	Molecular Formula	RT	Area %
1	Benzofuran, 6-ethenyl-4,5,6,7-tetrahydro-3,6-dime thyl-5-isopropenyl-, trans	C_15_H_20_O	12.64	3.66
2	4,4′-Dimethyl-2,2′-dimethylenebicyclohexyl-3,3′-diene	C_16_H_22_	15.16	8.43
3	Hexadecanoic acid, methyl ester	C_17_H₃₀O₂	20.74	50.73
4	9-Octadecenoic acid (Z)-, methyl ester	C_19_H_36_O_2_	23.21	27.17
5	Squalene	C_30_H_50_	32.56	9.13
6	Glycine, N-[(3à,5á)-24-oxo-3-[(trimethylsilyl)oxy]cholan-24-yl]-, methyl ester	C_30_H_53_NO_4_Si	33.68	0.88

## Data Availability

Data is contained within the article and supplementary material.

## Supplementary Materials

The following supporting information can be downloaded at: https://www.mdpi.com/article/10.3390/ph15091103/s1, Figure S1: The chromatogram of olive oil.; Figure S2: The chromatogram of palm oil.
