# Supplementary material for: The Impact of Dietary Consumption of Palm Oil and Olive Oil on Lipid Profile and Hepatocyte Injury in Hypercholesterolemic Rats
Source: Pharmaceuticals (Basel). 2022 Sep 4;15(9):1103. doi: 10.3390/ph15091103 (PMC9502270; doi:10.3390/ph15091103)
Supplement: Supplementary file 1 [file pharmaceuticals-15-01103-s001.zip › pharmaceuticals-1875270-supplementary.pdf]

Supplementary data

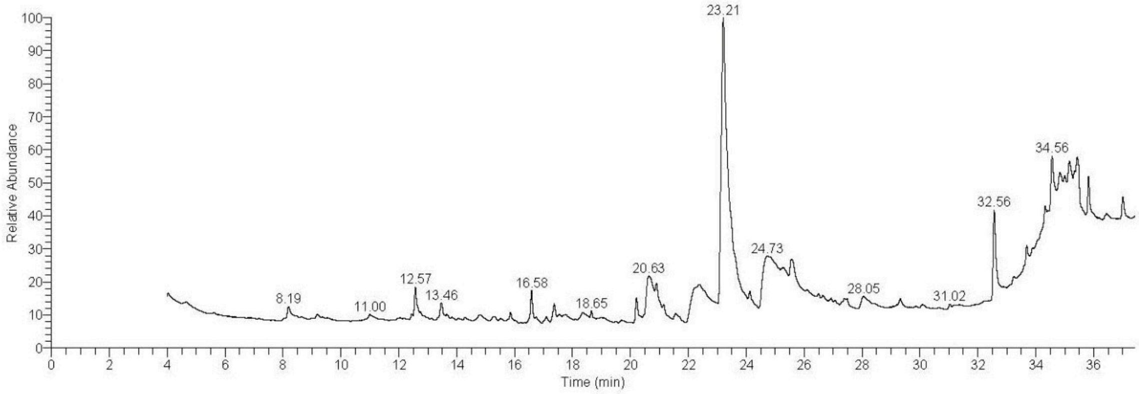

Figure S1. The chromatogram of olive oil.

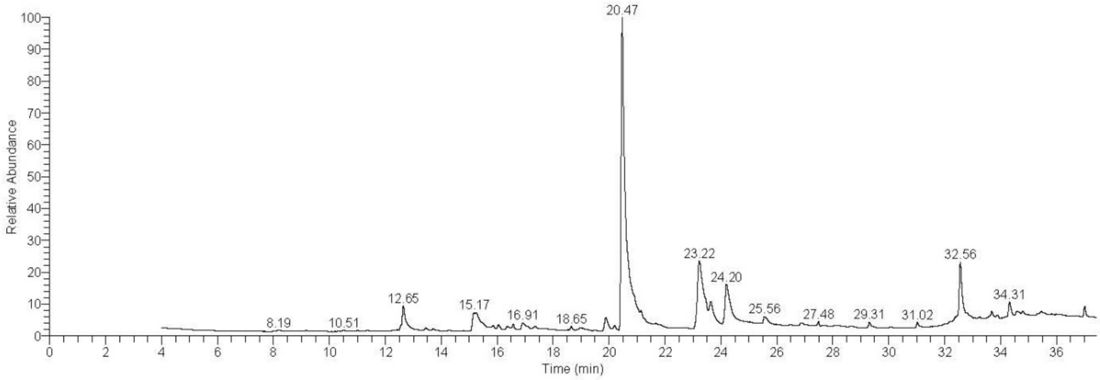

Figure S2. The chromatogram of palm oil.
